# Physical activity and academic procrastination in Chinese college students: The serial mediating roles of physical self-perceptions and self-esteem

**DOI:** 10.3389/fpsyg.2023.1083520

**Published:** 2023-03-10

**Authors:** Kai Ren, Xing Chen, Yanni Zhang, Fang Sun, Fan Peng

**Affiliations:** ^1^College of Physical Education and Health Science, Zhejiang Normal University, Jinhua, China; ^2^College of Physical Education, Chongqing Technology and Business University, Chongqing, China; ^3^Neuroscience and Mental Health Institute, University of Alberta, Edmonton, AB, Canada

**Keywords:** physical activity, physical self-perceptions, self-esteem, academic procrastination, serial mediating model

## Abstract

Studies have demonstrated that physical activity (PA) is negatively associated with academic procrastination. However, there is limited research on the mechanism underlying this relationship. This study aims to explore the relationship between PA and academic procrastination by investigating the roles of physical self-perceptions and self-esteem. 916 college students (650 females; *Mean* age = 19.11, *SD* age = 1.04) participated in the study. Participants completed the Physical Activity Rating Scale-3, the Physical Self-Perceptions Profile, the Rosenberg Self-Esteem Scale, and the Academic Procrastination Questionnaires. Descriptive statistics, Pearson’s correlation, and mediating effect analysis were carried out using SPSS 25.0. The results showed that (a) PA, physical self-perceptions, and self-esteem were negatively correlated with academic procrastination, (b) self-esteem mediated the association between PA and academic procrastination, and (c) physical self-perceptions and self-esteem sequentially mediated the association between PA and academic procrastination. These findings have deepened our understanding on the relationship between PA and academic procrastination, highlighting important approaches to deal with academic procrastination.

## 1. Introduction

Academic procrastination is a behavioral issue commonly seen among college students. As an intentional postponement in starting and/or finishing academic tasks or decision makings (Milgram et al., 1998), it is shown to be associated with several negative outcomes such as poor academic performance (Kim and Seo, 2015) and mental health conditions (Onwuegbuzie, 2004; Grunschel et al., 2012; Rice et al., 2012; Krause and Freund, 2014a,b). Given these adverse consequences, researchers have tried to identify factors that might reduce academic procrastination. Previous studies suggested that PA negatively correlates with general procrastination (Zhong and Chu, 2013; Codina et al., 2020; Shi et al., 2021) as well as academic procrastination (Ren et al., 2021). Although an association between PA and academic procrastination has been found, it is worth noting that the mechanism in which how PA influences academic procrastination have not been adequately studied among college students. Numerous studies have shown that low physical self-perceptions and self-esteem are common among physically inactive individuals (Fox, 2000; Legrand et al., 2020). Engaging in physical activities can increase feelings of physical competence and satisfaction with physical appearance, which leads to an increase in self-esteem (Jackson and Marsh, 1986). Results from studies indicate that individuals who participate in sports have higher levels of self-esteem, particularly in the physical domain (Taylor, 1995). As procrastination has been linked to the protection of a vulnerable sense of self-esteem (Steel, 2007), it appears that individuals with low self-esteem believe that any failure to meet standards highlights their inadequacy, causing them to avoid challenges by procrastinating (Rebetez et al., 2015). As a result, there could be a series of pathways in which low levels of PA lead to low physical self-perceptions, which in turn lead to lower self-esteem and eventually more frequent academic procrastination.

Based on the above, the present study aims to test the mediating roles of physical self-perceptions and self-esteem in the relationship between PA and academic procrastination.

### 1.1. Relationship between PA and academic procrastination in college students

PA has widely been viewed as a crucial part of a healthy lifestyle (Penedo and Dahn, 2005; Warburton et al., 2006). Risks of cardiovascular disease, all-cause mortality, and emotional problems (e.g., depression, anxiety) have been shown to be positively affected by regular PA (Lollgen et al., 2009; Rebar et al., 2015).

Several studies have suggested that PA can be an effective lifestyle intervention being economical and less dangerous, serving as an important strategy to prevent and treat mental health issues, and as a result, to improve life quality. Meanwhile, regular PA has also been found to benefit students’ academic behaviors that relates to procrastination. Studies have suggested that students who have a more active lifestyle (>150 min PA per week) showed less procrastination behaviors compared with their peers who do not have enough PA (Codina et al., 2020). It is demonstrated in previous studies that not only the duration of PA, the intensity level of it also plays a role in controlling procrastination behaviors (Zhong and Chu, 2013; Shi et al., 2021). For example, Shi et al. found that among the light, moderate and high levels of PA, the high level intense of PA leads to the least procrastination, followed by the moderate and light levels, which was also suggested in study conducted by Zhong and Chu in 2013. In this research, different aspects of PA which include duration, intensity as well as frequency were measured and their correlations with general procrastination have been found in Chinese college students. Similar results also echoed in another research that indicated PA promoted activities which features goal-achievement (e.g., completing a project) applying a daily diary approach (Young et al., 2018), and these activities are thought to be beneficial in students’ academic life.

Given a consistent result from previous studies showing that PA reduces the level of general procrastination, its effect on academic procrastination is likely to be existing but understudied. Therefore, the relationship between the PA and academic procrastination and the potential underlying psychological mechanisms needs to be further explored.

### 1.2. The mediating role of self-esteem

Self-esteem reflects the degree to which individuals recognize themselves positively (Rosenberg, 1965). Self-esteem is often seen positively related to PA (Herring et al., 2014; Arsandaux et al., 2020; Shang et al., 2021). The skill development hypothesis (Sonstroem, 1997a,b) proposes that self-esteem can be changed through experience, either positive or negative, development in skills, task mastery, success, etc. This hypothesis suggests that self-esteem is a result of involving in PA. Most cross-sectional and correlational studies support the positive relationship between PA and self-esteem. For instance, research has shown that individuals who participate in sports or are physically active have higher self-esteem compared to those who are inactive or do not participate in sports (Bowker, 2006). A positive correlation between physical self-perceptions and PA level has been observed in university students (Lindwall and Hassmen, 2004) and adults (Sonstroem et al., 1992). In a meta-analysis, Spence et al. focused specifically on studies examining PA and self-esteem in adults (Spence et al., 2005). The overall effect size was estimated to be 0.23, showing that PA significantly improved self-esteem in adults. In addition, Self-esteem is regarded as a predictor of academic procrastination (Zhang et al., 2018; Dike and Emmanuel, 2019). According to the Temporal Motivational Theory (TMT), self-esteem may act as a procrastination sensitivity trait that predicts academic procrastination (Steel et al., 2018). Research has shown that self-esteem has a negative impact on academic procrastination, as demonstrated by the significant negative correlation between these two variables. A study by Senécal et al. (1995) found that self-esteem negatively predicted procrastination in college students. This view has been supported by additional research findings (Dike and Emmanuel, 2019). These findings demonstrate the impact of self-esteem on academic procrastination. Meanwhile, a meta-analysis by Steel (2007) revealed a significant correlation between self-esteem and procrastination. In addition, a study by Klassen et al. (2008) found that self-esteem had a negative effect on academic procrastination in college students. These findings suggest that PA is related to self-esteem which is also correlated with academic procrastination. Therefore, we assume that self-esteem serves as a mediator between PA and academic procrastination.

### 1.3. The serial mediating roles of physical self-perceptions and self-esteem

Physical self-perceptions are defined as one’s perception or evaluation of their physical ability and physical appearance (Fox and Corbin, 1989). Physical self-perceptions have been presented as core features of the pursuit of mental health and well-being (Fox, 1998). Hence, approaches to foster positive physical self-perceptions have been widely called for. In this context, PA has been suggested as a significant and powerful instrument (Sonstroem, 1997a,b). Evidence shows that physical self-perceptions were identified as the predictor of self-esteem. As a sub factor of self-esteem, the development of physical self-perceptions was shown to enhance self-esteem (Fox and Corbin, 1989; Marsh et al., 2010). Students’ physical self-perceptions are indicated to influence their self-esteem (Garn et al., 2012; Harter, 2012). These findings imply that the change of individual’s physical self-perceptions could contribute to the overall self-esteem. In this sense, we assume that physical self-perceptions may improve the self-esteem, which may as a result improve academic procrastination.

Although some studies have revealed the effects of PA on academic procrastination, few studies have explored the mediating effect of physical self-perceptions and self-esteem in impacting academic procrastination. The above evidence provides an empirical basis for investigating the serial mediating effects of physical self-perceptions and self-esteem.

Therefore, this study will not only explore the direct correlation between PA and college students’ academic procrastination, but also explore the mechanism of the effect of PA on college students’ academic procrastination. Based on previous research in this area, this study hypnotizes that (a) self-esteem serves as a mediating variable between PA and academic procrastination. To be more specific, PA was positively correlated with self-esteem, and self-esteem was negatively correlated with academic procrastination, and (b) physical self-perceptions and self-esteem serve as serial mediating variables between PA and academic procrastination. To be more specific, PA were positively correlated with physical self-perceptions and self-esteem, and physical self-perceptions and self-esteem were negatively correlated with academic procrastination. This study aims to answer the question of how PA affects the academic procrastination in Chinese college students. Exploring the joint roles of physical self-perceptions and self-esteem between PA and academic procrastination in Chinese college students provide important implications that highlights the prevention of academic procrastination for college students.

## 2. Materials and methods

### 2.1. Participants and ethnics’ statement

A total of 916 students were recruited by convenient sampling from a university located in Zhejiang Province (China). They signed a written informed consent form before the survey and received no compensation after the survey completion. Data were collected by filling out a series of paper-based questionnaires about demographic characteristics, the Physical Activity Rating Scale-3 (PARS-3), the Physical Self-Perceptions Profile (PSPP), the Rosenberg Self-esteem Scale (RSES) and Academic Procrastination Questionnaire for College Students (APC) under the guidance of trained assistants. All processes were carried out in accordance with the guidelines of the Ethics Committee of Zhejiang Normal University and the Declaration of Helsinki, and this study was approved by the Ethics Committee of Zhejiang Normal University. Participants aged 18–22 years old (Mean = 19.11, *SD* = 1.04), which included 650 females and 266 males. The sample consisted of 456 freshmen, 318 sophomores, and 142 juniors.

### 2.2. Measures

#### 2.2.1. Physical activity

The Physical Activity Rating Scale-3 (PARS-3) which was revised by Liang (1994) was applied to assess participants’ physical activity. It is a 3-item self-reported scale which contains physical activity intensity, physical activity time, and physical activity frequency. The scale was a 5-point Likert scale, with each item scored 1–5. The total physical activity scores = intensity score × (activity time score 1) × frequency score (scored 0–100).

#### 2.2.2. Physical self-perceptions

The self-report questionnaire Physical Self-Perceptions Profile (PSPP) (Fox and Corbin, 1989; Xu and Yao, 2001) was used to measure physical self-perceptions. The PSPP contains 30 items which covers five sub-domains, with 6 items in each domain. These sub-domains include perceptions in a. Sport Competence (Sport), b. Physical Conditioning (Condition), c. Bodily Attractiveness (Body), d. Physical Strength (Strength), and e. Physical Self-Worth (PSW). Individuals specify their responses to each item to 4 points (i.e., 1 to 4). The results showed that the scale had good structural validity:

Sport Competence: *χ*^2^/df = 1.707, RMSEA = 0.028, CFI = 0.997, NFI = 0.993.

Physical Conditioning: *χ*^2^/df = 1.533, RMSEA = 0.024, CFI = 0.999, NFI = 0.997.

Bodily Attractiveness: *χ*^2^/df = 2.114, RMSEA = 0.035, CFI = 0.998, NFI = 0.996.

Physical Strength: *χ*^2^/df = 3.887, RMSEA = 0.056, CFI = 0.976, NFI = 0.968.

Physical Self-Worth: *χ*^2^/df = 4.100, RMSEA = 0.058, CFI = 0.992, NFI = 0.990.

In this study, the Cronbach’s alpha was 0.94.

#### 2.2.3. Self-esteem

The Rosenberg Self-esteem Scale (RSES) was used to evaluate global self-esteem (Rosenberg, 1965). The RSES is a 10-item scale with five positively and five negatively worded items using a Likert scale ranging from 1(strongly disagree) to 4 (strongly agree). Higher scores indicate higher levels of global self-esteem. The results showed that the scale had good structural validity: *χ*^2^/df = 3.895, RMSEA = 0.056, CFI = 0.998, NFI = 0.997. In the current study, the Cronbach’s alpha was 0.86.

#### 2.2.4. Academic procrastination

Academic procrastination was assessed by the 26-item Academic Procrastination Questionnaire for College Students (APC), which was revised by Han (2008). Participants were requested to answer the degree of their academic procrastination. Each item was assessed by a five-point scale ranging from 1 (never) to 5 (always). The total score of all items represents the levels of academic procrastination, and higher scores reflect higher levels of academic procrastination. The results showed that the scale had good structural validity: *χ*^2^/df = 3.755, RMSEA = 0.055, CFI = 0.937, NFI = 0.917. In this study, Cronbach’s alpha was 0.80.

### 2.3. Data analysis

Data in this study were analyzed by SPSS Version 25.0. Pearson correlation analysis was adopted to evaluate the association among variables. Hayes (2013) SPSS macro-PROCESS 3.3 (model 6) with 95% percentile confidence interval (CI) based on 5,000 bootstrap samples was used to examine the indirect effects of PA and academic procrastination through physical self-perceptions and self-esteem. The indirect effect is considered statistically significant if the CI does not contain zero.

To assess the validity of the questionnaire for the sample used in this study, Confirmatory Factor Analysis (CFA) was performed using Amos 24.0. Only PSPP, RSES, and APC were tested since physical activity is an observed variable and the PARS-3 was excluded from the CFA. The construct validity was evaluated using statistical indices including CMIN/DF, RMSEA, NFI, and CFI. A good fit of the model is indicated by RMSEA values of 0.08 or lower, and values of 0.95 or higher for GFI, NFI, and CFI (Meyers et al., 2017).

## 3. Results

### 3.1. Common method biases test

The self-reported data in this study may have potential biases, so a Harman’s single factor test was conducted to check for any common methodological biases before the data analysis (Podsakoff et al., 2003; Zhou and Long, 2004). The results of the exploratory factor analysis showed that there were 14 factors with eigenvalues greater than 1 and the first common factor accounted for 21.92% of the variance, indicating that there were no significant common biases in the study (Xiong et al., 2012). To enhance the reliability of the findings, it is recommended to consider using multiple methods to collect data and to validate the results with further studies.

### 3.2. Descriptive statistics and bivariate correlation analysis

Table 1 shows means, standard deviations, and Pearson’s zero-order correlation for the variables. Correlation analysis showed that PA was positively associated with physical self-perceptions and self-esteem (*r* = 0.28, *p* < 0.01; *r* = 0.34, *p* < 0.01, respectively). Likewise, physical self-perceptions were also positively associated with self-esteem (*r* = 0.42, *p* < 0.01). In addition, PA, physical self-perceptions, and self-esteem were negatively correlated with academic procrastination (*r* = −0.17, *p* < 0.01; *r* = −0.31, *p* < 0.01; *r* = −0.41, *p* < 0.01, respectively). The inter-correlations among the variables provided initial support to the hypothetical indirect effects.

**Table 1:** Descriptive statistics and correlations among variables (*N* = 916).

Variable	Mean	SD	1	2	3	4
1. Physical activity	24.72	23.27	–			
2. Physical self-perceptions	2.12	0.48	0.28**	–		
3. Self-esteem	3.00	0.45	0.34**	0.42**	–	
4. Academic procrastination	2.83	0.55	−0.17**	−0.31**	−0.41**	–

**p < 0.01.

### 3.3. Regression analysis and serial mediation analysis

Table 2 shows the results of the regression analysis. The results indicated that PA significantly predicted physical self-perceptions (*β* = 0.28, *p* < 0.001). In the second step of the regression analysis, PA and physical self-perceptions were added as predictors. Both PA and physical self-perception significantly predicted self-esteem (*β* = 0.11, *p* < 0.001; *β* = 0.36, *p* < 0.001, respectively). In the third step, PA, physical self-perceptions, and self-esteem were added to the regression model, it was found that both physical self-perceptions and self-esteem significantly predicted academic procrastination (*β* = −0.17, *p* < 0.001; *β* = −0.34, *p* < 0.001; respectively), while the relationship between PA and academic procrastination was not statistically significant anymore. Based on the research findings above, PA plays a full mediating effect on academic procrastination.

**Table 2:** Results of regression analysis.

Regression equation		Fitting indices		Regression coefficient	
Outcome variables	Predictor variables	*R^2^*	*F*	*β*	*t*
Physical self-perceptions		0.08	74.60***		
	PA			0.28	8.64***
Self-esteem		0.23	134.87***		
	PA			0.11	3.21**
	Physical Self-perceptions			0.36	10.60***
Academic procrastination		0.19	56.30***		
	PA			−0.01	−0.34
	Physical Self-perceptions			−0.17	−5.01***
	Self-esteem			−0.34	−9.88***

**p < 0.01, ***p < 0.001. PA, physical activity.

The serial mediation model of the relationship between PA and academic procrastination by and self-esteem is presented in Table 3. The direct effect of PA on academic procrastination (*β* = −0.007, Boots95%*CI* [−0.044, 0.032], *p* > 0.05) did not appear to be significant after the mediators (physical self-perceptions and self-esteem) were added in the model. The total indirect effect of PA on academic procrastination through physical self-perceptions and self-esteem was −0.097 (Boots 95% *CI* [−0.124, −0.073], *p* < 0.001), from which the indirect effects of PA on academic procrastination through self-esteem was −0.050 (Boots 95% *CI* [−0.066, −0.035], *p* < 0.001),which accounted for 48.08% of the total effect; the serial mediating effect through physical self-perceptions and then self-esteem was −0.020 (Boots95% *CI* [−0.028, −0.013], *p* < 0.001), which accounted for 19.23% of the total effect. The total mediating effect was 93.27% of the total effect. These results demonstrated significant mediating effects of physical self-perceptions and self-esteem between PA and academic procrastination.

**Table 3:** Total, direct, and indirect effects of physical activity (X) on academic procrastination (Y) through physical self-perceptions (M1) and self-esteem (M2) (*N* = 916).

Effect	Point estimate	*SE*	Boots 95%*CI*	
			Lower	Upper
Total effects	−0.104	0.020	−0.143	−0.064
Direct effect	−0.007	0.020	−0.044	0.032
Total indirect effects	−0.097	0.013	−0.124	−0.073
X → M2 → Y	−0.050	0.008	−0.066	−0.035
X → M1 → M2 → Y	−0.020	0.004	−0.028	−0.013

CI, Confidence Interval.

### 3.4. *R^2^* (explained variance) and *f^2^* (effect extent)

The structural model was evaluated based on the *R^2^* (coefficient of determination) and *f^2^* (effect size) criteria (Chin, 1998). *R^2^* measures the proportion of variance in the dependent variable that is explained by the independent variables in the model, with a higher value indicating a better fit. The *f^2^* measures the practical significance of the model, with higher values indicating a larger contribution of the independent variables to the dependent variable. The *R^2^* of 0.19, 0.33, and 0.67 would be considered weak, moderate, and substantial, respectively. The *f^2^* of 0.02, 0.15, and 0.35 would be considered small, medium, and high, respectively. These criteria were used to assess the quality and validity of the structural model.

**Figure 1:**
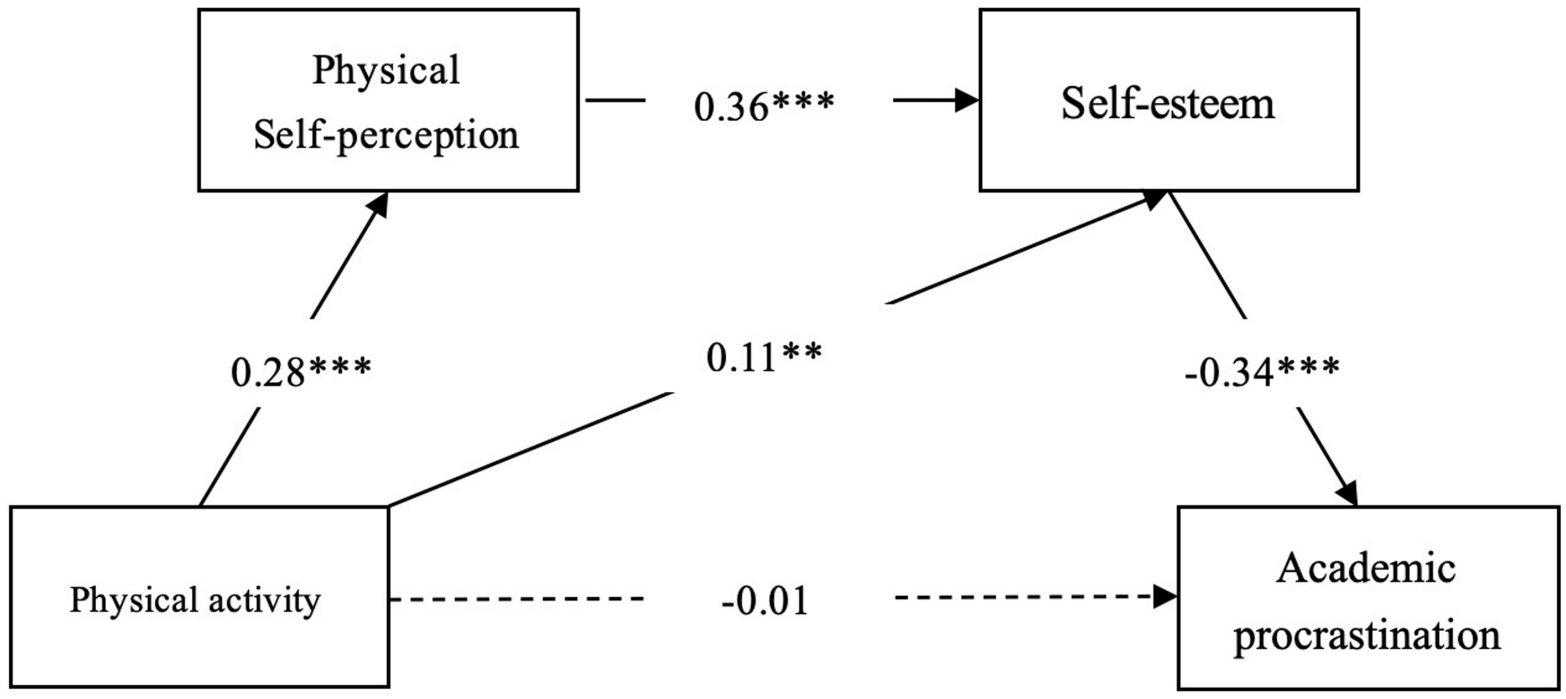
Serial mediation model of physical activity and academic procrastination through physical self-perceptions and self-esteem as serial mediators. *p < 0.05, **p < 0.01, ***p < 0.001.

The results of this study suggest that PA has a moderate impact on physical self-perceptions and self-esteem, but their effect on academic procrastination is only small. The overall model had a weak impact with an *R^2^* value of 0.19. The medium effect sizes (*f^2^*) were evaluated for the associations between PA and physical self-perceptions (0.28), PA and self-esteem (0.21), and PA and academic procrastination (0.23). The small effect sizes (*f^2^*) were detected for the relationships between physical self-perceptions and self-esteem (0.13) and self-esteem and academic procrastination (0.09). These findings suggest that PA may have a greater impact on physical self-perceptions and self-esteem compared to academic procrastination (Figure 1; Table 4).

**Table 4:** *R^2^* and *f^2^* findings.

Paths	Path coefficients	*f^2^*	*R^2^*
PA → PSP	0.28	0.28	0.08
PA → SE	0.11	0.21	0.23
PSP → SE	0.36	0.13	
PA → AP	−0.01	0.23	0.19
SE → AP	−0.34	0.09	

PA, Physical Activity; PSP, Physical Self-Perceptions; SE, Self-Esteem; AP, Academic Procrastination.

## 4. Discussion

In this study, we investigated the mediating effects of physical self-perceptions and self-esteem in the relationship between PA and academic procrastination. Our regression analysis reveals a serial mediating model of physical self-perceptions and self-esteem in this association. According to our findings, PA negatively predicted academic procrastination, which indicates that physically active undergraduates are less likely to experience academic procrastination and it echoed the results from previous studies (Zhong and Chu, 2013; Ren et al., 2021; Shi et al., 2021). One of the potential reasons is that PA significantly increases positive emotions, which promotes better performance and less procrastination in academic-related activities (Carmona-Halty et al., 2021). Meanwhile, significant pairwise correlations among PA, physical self-perceptions, self-esteem, and academic procrastination are found in our correlation analysis, which also supported our hypotheses.

### 4.1. The mediating role of self-esteem

Our findings revealed that self-esteem played a mediating role in the relationship between PA and academic procrastination. Thus, hypothesis H1 was supported. The positive impact of PA on self-esteem was consistent with previous evidence (Ekeland et al., 2005; Spence et al., 2005; Joseph et al., 2014) and the skill-development hypothesis (Sonstroem, 1998). The skill-development hypothesis suggests that perception of success and being rewarded booster individuals’ self-evaluation and their perceived competence. In the context of PA, the skill-development hypothesis advised that enhancements in motor kill that arise from participating in PA trigger the improvement of self-esteem, which is considered as an inherent outcome of successful mastery of motor skills (Sonstroem, 1998). Furthermore, college students who were physically inactive were more likely to experience academic procrastination due to low self-esteem. Why is self-esteem a predictor of academic procrastination? It actually echoes several psychological theories. First, TMT states that the gap between intention and action is the primary cause of procrastination (Klingsieck, 2013; Steel et al., 2018). People with low self-esteem often have a lower sense of task efficacy (Huang, 2010; Yerdelen et al., 2016) and are more susceptible to distractions, leading to a lower intention to complete tasks and a longer gap between intention and action, resulting in more frequent procrastination. Second, the self-determination theory (Deci and Ryan, 1985, 2008) suggests that the perception of competence is a basic need that motivates task completion (Codina et al., 2018). People with low self-esteem may lack the competence to initiate tasks and thus may fail to complete tasks on time, resulting in academic procrastination. Third, the self-efficacy theory (Bandura, 1977) states that self-efficacy is the individual’s confidence in their ability to successfully engage in certain behaviors. Individuals with low self-esteem often have lower self-efficacy (Gowan et al., 2000; Judge and Bono, 2001) and a lack of confidence in undertaking academic tasks, leading to academic procrastination. Therefore, it is suggested that interventions to improve the academic performance of students with procrastination should focus on improving their level of self-esteem (Duru and Balkis, 2017).

### 4.2. The serial mediating roles of physical self-perceptions and self-esteem

Beyond above, a serial mediating effect has also been found in this study by physical self-perceptions and self-esteem. In other words, hypothesis H2 was supported. Self-esteem is strongly influenced by one’s physical self-perceptions which encompasses 4 subdomains including sport competence, body attractiveness, physical strength, and physical conditioning. These subdomains have all been reported to associate with self-esteem and among them the prediction of bodily attractiveness was the strongest (Lindwall et al., 2011). For the influence of PA on physical self-perceptions, it was reported in previous research that more frequent and longer duration of PA lead to a higher level of physical self-perceptions (Lindwall and Hassmen, 2004), which has also been found in our study.

Self-enhancement hypothesis suggests that individuals’ perception of themselves is fundamental to their actions. Individuals tend to engage in activities that they believe will lead to success, thus enhancing self-esteem (Sonstroem, 1998). This view supports the intention of human behavior to preserve or confirm self-judgments and expectations. Thus, students with higher perceived competence are predicted to be more likely to participate in academic training and activities than those with lower perceived competence. These findings demonstrate that undergraduate students with higher PA are more likely to have higher levels of physical self-perceptions, which in turn leads to higher levels of self-esteem, and eventually leads to decreased risk of academic procrastination. In summary, regular PA is highly recommended in undergraduate students for affecting their physical self-perceptions, followed by improved self-esteem, which will eventually reduce their academic procrastination behaviors.

### 4.3. Theoretical and practical implications

The results of this study have significant theoretical and practical implications in education. Theoretically, it provides a new perspective on the relationship between PA and academic procrastination by examining the role of physical self-perceptions and self-esteem. The findings add to the existing literature and offer a unique understanding of the mechanism by which PA influences academic procrastination in college students. The results also show that the three theories used in this study (TMT, Self-enhancement hypothesis, and Skill Development hypothesis) each offer valuable insight and that a unified model incorporating all three theories provides a comprehensive explanation of PA’s effect on academic procrastination. These results highlight the importance of incorporating multiple theories in exploring academic procrastination.

The practical implications of the study suggest that academic procrastination in college students may be linked to low physical self-perceptions and self-esteem. Hence, PA interventions could be an effective way to prevent and reduce academic procrastination. Educators in colleges should therefore prioritize promoting PA among students and finding ways to encourage students to be more physically active. This may lead to improved physical self-perceptions and self-esteem, which in turn could result in decreased academic procrastination.

### 4.4. Limitations

Nevertheless, this study has several limitations. First, as a cross-sectional study design, it is difficult to clarify whether there are causal relationships among variables. Longitudinal data are still needed for further studies. Second, the data of the current study came from self-report questionnaires, which might suffer from potential bias. Third, given our specific study population, cautions should be taken to generalize our findings. More research from other populations needs to be done in the future. Third, it is important to note that the results of this study are based on a sample of Chinese college students, with a majority of female participants (70.96%). This limits the generalizability of the findings to other cultures or populations. It is advisable for future studies to use samples with a more equal representation of both genders and a more diverse range of populations. This would increase the robustness of the findings and ensure that the results are more applicable to a wider range of individuals.

## 5. Conclusion

The present study provides empirical evidence of the mechanisms linking PA to academic procrastination. PA alleviates academic procrastination, in which not only simply mediated by self-esteem, but also mediated by physical self-perceptions and self-esteem serially. These findings emphasize the significance of physical self-perceptions and self-esteem when exploring the mechanisms through which PA relates to academic procrastination. Therefore, PA interventions that aim at decreasing academic procrastination could benefit from combing with a multifaceted method therapeutically targeting the promotion of physical self-perceptions and self-esteem. Future studies are needed to examine the mediating effects of physical self-perceptions and self-esteem between PA and academic procrastination in broader populations.

## Data availability statement

The raw data supporting the conclusions of this article will be made available by the authors, without undue reservation.

## Ethics statement

The studies involving human participants were reviewed and approved by Zhejiang Normal University. The patients/participants provided their written informed consent to participate in this study.

## Author contributions

KR, XC, FS, and FP: conceptualization. KR, YZ, FS, and FP: methodology. YZ: software. FS and FP: validation. KR: formal analysis, writing—original draft preparation, and funding acquisition. XC: data curation. FP: writing—review and editing. XC: visualization and project administration. All authors have read and agreed to the published version of the manuscript.

## Funding

This study was supported by the National Social Science Foundation of China (Grant No. 19BTY079).

## Conflict of interest

The authors declare that the research was conducted in the absence of any commercial or financial relationships that could be construed as a potential conflict of interest.

## Publisher’s note

All claims expressed in this article are solely those of the authors and do not necessarily represent those of their affiliated organizations, or those of the publisher, the editors and the reviewers. Any product that may be evaluated in this article, or claim that may be made by its manufacturer, is not guaranteed or endorsed by the publisher.
